# Research on the Composition and Manufacturing Technology of the Single-Eared Octagonal Gold Cup Unearthed from the Turki Mountain Tomb in Inner Mongolia

**DOI:** 10.3390/ma19143082

**Published:** 2026-07-17

**Authors:** Yawei Zhang, Lijuan Dong, Weidong Hu, Lei Yang, Li Li

**Affiliations:** 1 School of Yungang Ology, Shanxi Province Key Laboratory of Microstructure Electromagnetic Functional Materials, Institute of Solid State Physics, Shanxi Datong University, Datong 037009, China; zhangyw202110@163.com; 2 Beijing Key Laboratory of Millimeter Wave and Terahertz Technology, Beijing Institute of Technology, Beijing 100081, China; 3 Shanxi Key Laboratory of Advanced Semiconductor Optoelectronic Devices and Integrated Systems, Jincheng Research Institute of Opto-Mechatronics Industry, Jincheng 048000, China; yangleidtdx@163.com (L.Y.); lili@jcgjd.org.cn (L.L.)

**Keywords:** Turki Mountain Tomb, single-eared octagonal gold cup, manufacturing technology, composition analysis, microscopic observation

## Abstract

The Turki Mountain Tomb, one of the three most representative Liao Dynasty tombs, has yielded numerous exquisite gold and silver artifacts during excavation that have drawn global attention for their superb craftsmanship and distinctive ethnic and period characteristics. However, their manufacturing technology had seldom been studied. In this paper, alloy composition analysis and surface microscopic observation were performed on the single-eared octagonal gold cup unearthed from the Turki Mountain Tomb, utilizing portable X-ray fluorescence spectroscopy (p-XRF) and an ultra-depth field microscope. The composition results at different base material locations of the gold cup were similar, with gold content ranging from 84% to 88% and silver content ranging from 10% to 13%. The p-XRF spectra at the exact center of the ring foot, as well as the pearl roundel on the abdominal ridge and rim, showed dominant Au with minor Ag content. Therefore, it could be concluded that the material of the gold cup was made of Au-Ag alloy. Microscopic observation preliminarily revealed that the manufacturing process involved casting, engraving, and welding. The single-eared octagonal gold cup exhibited numerous conspicuous shrinkage cavities, and it was inferred that the gold cup was formed using casting technology. After the cup body, ring foot, and finger pad were cast separately, they were welded together to form the complete gold cup. In addition, green solder and insufficient fusion of welding material were found between the weld seam of the cup body and the ring foot. The exterior surface of the gold cup was adorned with patterns, such as fish-toe circle, upward lotus motif, and pearl roundels. The average diameter of the fish-toe circle was 303 μm. By examining the overlapping conditions of engraving, it could be inferred that the proposed engraving sequence was to engrave the fish-toe circle first, followed by the flower patterns. As a representative of the exquisite artifacts from the Turki Mountain Tomb, the research of the composition and manufacturing technology of the gold cup provides reference data for the scientific analysis of Liao Dynasty gold and silver artifacts.

## 1. Introduction

The Liao Dynasty (916–1125 AD) was established by the ancient Khitan people in China. It had nine emperors and lasted for 209 years. During this period, the rulers adopted a culturally inclusive policy that built upon the heritage of previous northern nomadic cultures, fostering a distinctive national culture. The Turki Mountain Tomb is an early aristocratic tomb of the Liao Dynasty. The numerous cultural relics unearthed have greatly enriched our understanding of the Liao Dynasty. The excavation provided fresh materials for Liao Dynasty archaeology and holds great importance for research on its politics, economy, culture, art, clothing, daily customs, and funeral system [1]. Since ancient times, gold and silver have long served as markers of identity and social standing, and the Liao Dynasty was no exception. Liao Dynasty gold and silver artifacts stand out for their exquisite craftsmanship, blending foreign and local cultural elements to fully embody the aesthetic of ancient artifacts. A wide variety of gold and silver wares were unearthed from the Liao Dynasty, mainly including tableware, ornaments, daily utensils, horse gear, and burial objects. During this period, a policy of lavish burial was practiced, and the demand for gold and silver objects among the princes and nobles promoted the maturity of gold and silver ware manufacturing technology. Gold and silver wares of the Liao Dynasty were still considered luxury items, mainly used by the nobility. Evidence from excavated tombs shows that gold and silver wares found in aristocratic tombs vastly outnumber those in commoner graves. The nobility not only possessed these items in overwhelming quantities but also owned pieces of distinctly superior manufacturing technology. The development of gold and silver ware craftsmanship in the Liao Dynasty was inseparable from factors such as the supply and smelting of raw materials, government support, and the aristocrats’ pursuit of luxury items. These elements together brought about the superb metalworking techniques of the Liao Dynasty.

**Figure 1:**
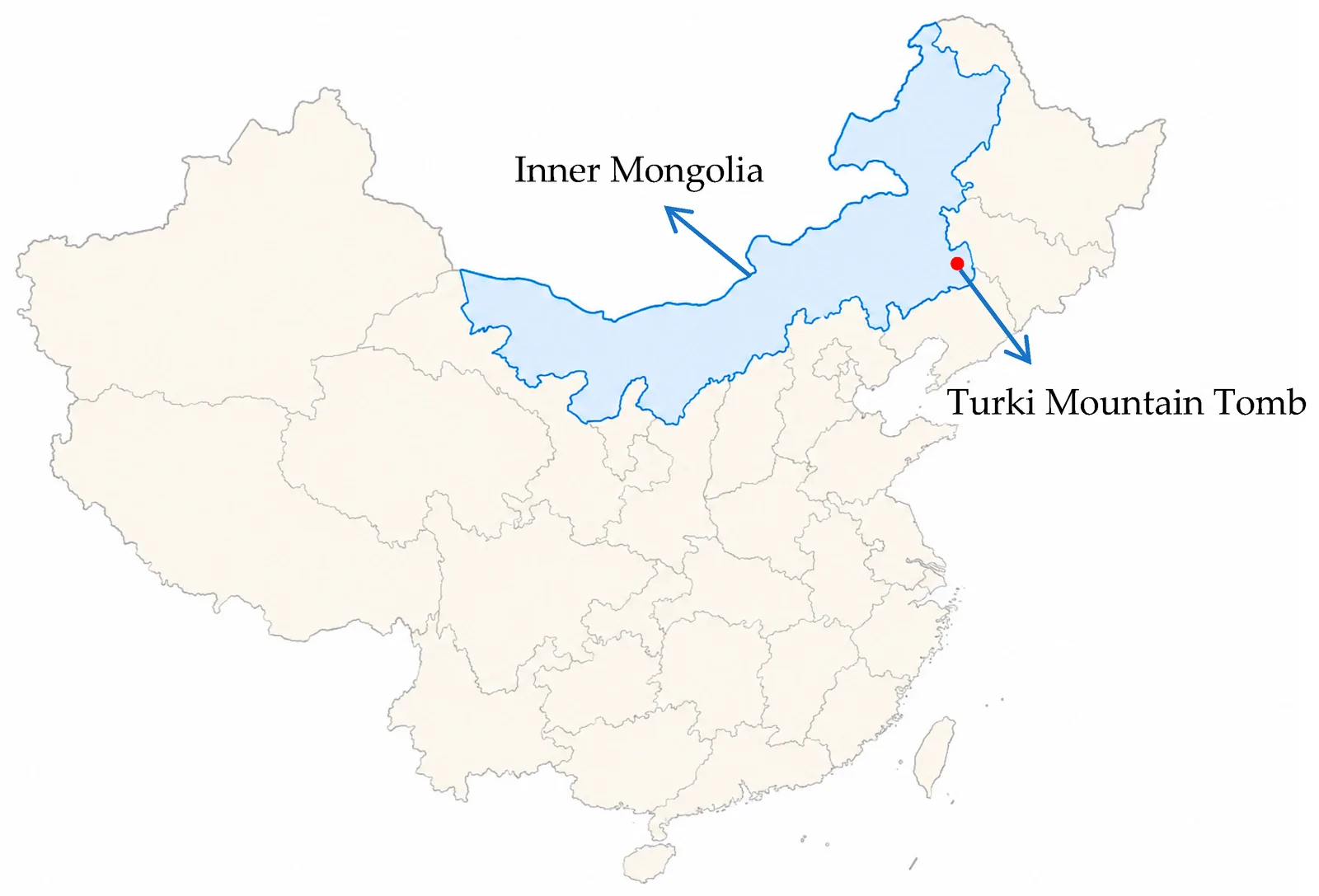
Geographical location map of the Turki Mountain Tomb.

In March 2003, a tomb was accidentally discovered during quarrying activities at the Turki Mountain quarry site. Subsequently, the Inner Mongolia Region Institute of Cultural Relics and Archaeology, Tongliao City Museum, and other institutions established an archaeological team and promptly commenced a formal excavation of the tomb [1]. The Turki Mountain Tomb is situated on the southeastern slope of the Great Turki Mountain in Horqin Left Rear Banner, Tongliao City, Inner Mongolia, about 50 km from Tongliao City (Figure 1). This tomb is a large stone chamber tomb of the Khitan people from the Liao Dynasty, consisting of a tomb passage, a tomb door, a passageway, a tomb chamber, and left and right ear chambers. The tomb passage is a long sloping passage, measuring 48 m, with the side walls constructed of stone blocks and black clay. There is a sealing stone at the entrance of the tomb, and on the inside there is a pair of wooden doors. The tomb chamber and the tomb door are connected by a passageway. The tomb chamber is nearly square, with left and right ear chambers at the front of the tomb chamber, which are rectangular in shape. A large number of precious cultural relics were unearthed from this tomb. The unearthed burial objects include a painted wooden coffin, inner coffin, and coffin platform. Notably, the painted wooden coffin and the coffin platform are the first complete examples ever discovered in Inner Mongolia. The painted wooden coffin is predominantly colored in red and black, with carved patterns such as cranes, phoenixes, and other motifs. The excavation of the tomb took place from March to May in 2003. The tomb unearthed a variety of cultural relics, including over 200 items such as bronze, gold, silver, wood, glassware, harnesses, lacquer, and silk. The discovery of the Turki Mountain Tomb represents another major archaeological find in Inner Mongolia, following the earlier discoveries of the Tomb of State Chen Princess and the Tomb of Yelu Yuzhi. It represented an aristocratic tomb from the early Liao Dynasty, and this archaeological discovery was honored as one of the top ten archaeological discoveries in China in 2003.

The funeral system of the Khitan nobility in the Liao Dynasty was smaller in scale than that of the emperor. They implemented a policy of lavish burial, often using tableware as burial objects and sacrificing animals such as horses and sheep [2]. The gold cup buried embodies the Liao Dynasty notion of treating the dead as if they were alive, indicating that people at that time believed the afterlife still required the cherished objects they valued during their lifetime. The single-eared octagonal gold cup is key physical evidence of the superior noble tableware in the early Liao Dynasty and a symbol of the wealth and social standing of the noble tomb owners in the Liao Dynasty. The single-eared octagonal gold cup is extremely similar in shape to the Sogdian silver cup, having a handle, with both being octagonal cups. The shape of the gold cup belongs to the typical Sogdian vessel form. It has a loop handle attached to one side, and the upper part of the handle is fitted with a finger pad, which is wider than the cross-section of the handle, and the lower side is decorated with small protrusions. The single-eared octagonal gold cup is similar in shape to the Sogdian silver cup, indicating that it was influenced by the Sogdian silver cup. The gold cup exhibited a form with vertical ridges decorated with pearl roundels, which represented a foreign cultural element. During the gradual Sinicization of gold and silver wares, the Han people in the Tang Dynasty largely did not use this shape in their gold and silver wares, but the custom of using this shape still survived in the Liao Dynasty [3]. The pearl roundel of the Liao Dynasty is more rounded and fuller than that of the Tang Dynasty. Such a pattern is decorated on the rim, on the abdomen ridge, and on the ring foot. This decorative style was influenced by Western cultures. The pattern of the gold cup is different from that of the Sogdian silver cup. The abdomen surface is a separated individual image, featuring traditional Chinese animal, plant, and human figures, which belonged to traditional Chinese patterns. Therefore, it could be determined that the gold cup was an imitation of Sogdian vessels. The shape and pattern of the gold cup integrated elements of Central Plains culture and Sogdian, Persian, and other cultures, serving as direct physical evidence of the interaction and integration of different cultures. The sample selected for this study was a single-eared octagonal gold cup from among the gold artifacts. Due to distinctive period and regional features for the single-eared octagonal gold cup, effective testing and analysis can provide scientific evidence for the manufacturing technology and technological inheritance of Liao Dynasty gold and silver artifacts and offer important recommendations for subsequent preventive conservation and restoration.

Current research on gold and silver artifacts of the Liao Dynasty has explored their periodization, typology, pattern evolution, artistic styles, cultural interactions, functions, and cultural connotations, drawing upon archaeology, history, fine arts, and folklore [2,3,4]. The theoretical research on the manufacturing technology of Liao Dynasty gold and silver artifacts was fragmented and unsystematic, with descriptions often being superficial and limited to small sections of scholarly articles [5,6]. For example, ref. [7] categorically explored manufacturing technology of Liao Dynasty gold and silver artifacts based on prior research and also compared them with Tang Dynasty manufacturing technology, all within the same theoretical framework.

With the continuous upgrading of scientific instruments and equipment, scholars currently utilize various testing methods such as an X-ray diffractometer (XRD) [8], XRF [9,10], metallographic analysis [11], optical microscope [12], scanning electron microscopy with energy dispersive spectroscopy (SEM-EDS) [12,13,14], and Monte Carlo simulation methods [15] to investigate the composition, the cross-section, and internal structure of gold and silver artifacts. These basic data provided an essential reference for further analysis of various gold and silver artifacts. At present, the academic community had made some progress in detecting the manufacturing technology of gold and silver artifacts [16,17,18]. For instance, ref. [19] comprehensively analyzed the decorative features, materials, and manufacturing technology of the gold- and silver-inlaid iron sticks from Xuzhou Tushan Tomb No. 2 using multiple methods (X-ray imaging, super depth-of-field microscope, metallographic analysis, and SEM-EDS) and detailed the morphology and layout of the surface decorations. Similarly, ref. [20] used microscopy and SEM-EDS to study a delicate silver cosmetics box from a Tang Dynasty tomb at the Xiaolizhuang site, revealing the sophisticated techniques in making the artifact. In addition, ref. [21] used non-destructive methods such as microscopy, X-ray imaging, SEM-EDS, and XRD to examine the morphology and elemental composition of gold jewelry, including a finger ring and an earring with exotic features. As evident from the aforementioned literature, scholars have utilized non-destructive methods such as X-ray imaging, microscopy, SEM-EDS, and XRD to examine gold and silver artifacts. These studies have enhanced our understanding of ancient gold and silver artifact manufacturing technology.

However, there is still a gap in the non-destructive testing of manufacturing technology for Liao Dynasty gold and silver artifacts from the Turki Mountain Tomb in Inner Mongolia. In this paper, a specific non-destructive testing case analysis was conducted on the single-eared octagonal gold cup from the Turki Mountain Tomb. Using the single-eared octagonal gold cup as the research object, this study utilized portable X-ray fluorescence spectroscopy (p-XRF) and an ultra-depth field microscope to perform alloy composition analysis and surface microscopic observation of the gold cup. This study aims to determine the composition and main manufacturing technology of the gold cup, reconstruct its entire production process, and clarify its technological features. The findings serve as a key reference for other gold and silver artifacts from the Turki Mountain Tomb and provide reliable data for future cultural relics protection.

## 2. Materials and Methods

### 2.1. Sample Information

**Figure 2:**
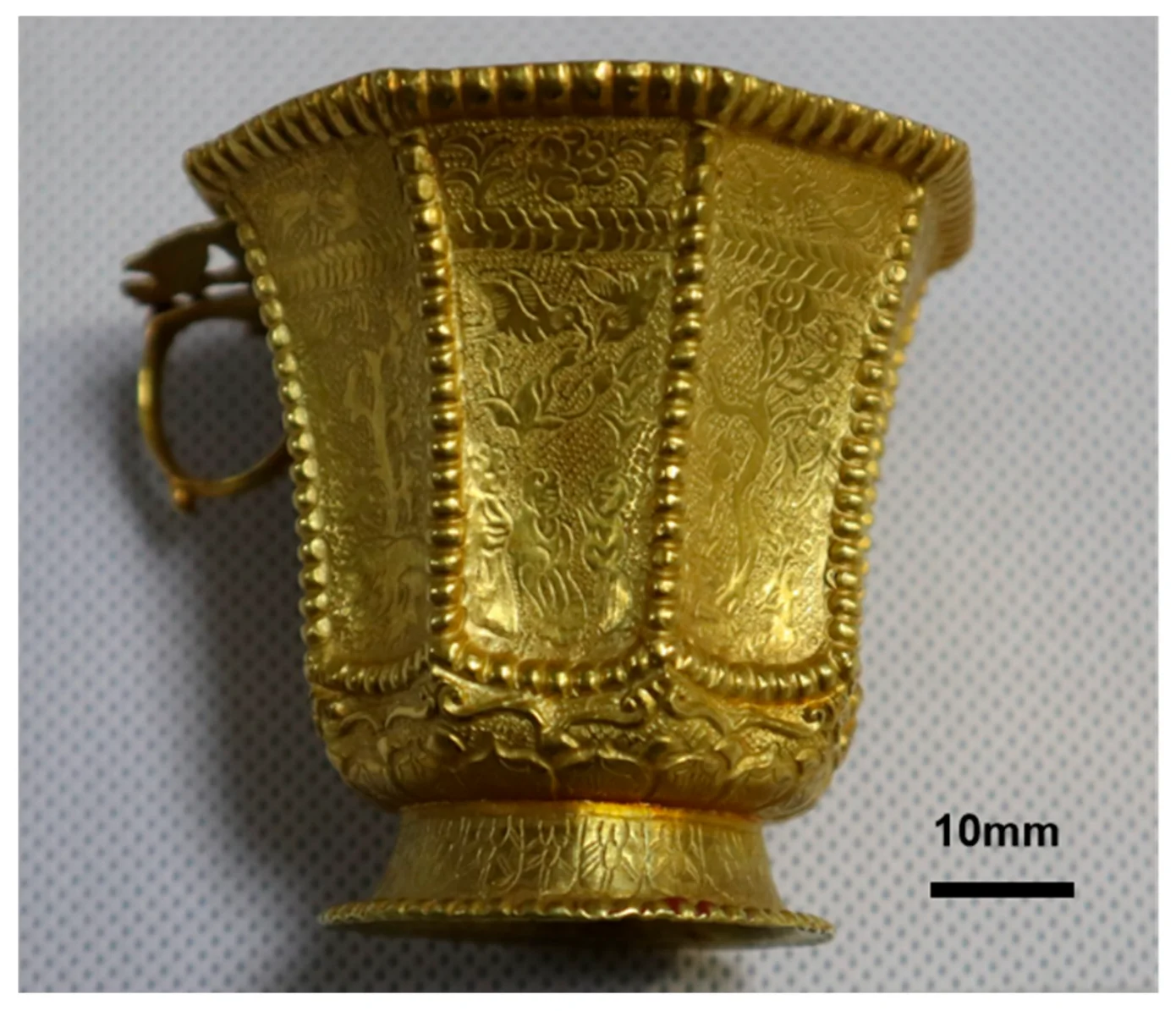
Photo of the single-eared octagonal gold cup.

The single-eared octagonal gold cup (Figure 2) was excavated from the Turki Mountain Tomb. It has a weight of 67.004 g, a height of 57.98 mm, and a rim diameter of 62.4 mm. The gold cup is wide-mouthed, with a slightly inward-curved wall, octagonal body, trumpet-shaped ring foot, fish-tail-shaped finger pad, and leaf-bud-shaped wing handle. The rim, abdomen ridge, and base edge of the ring foot are decorated with pearl roundels [22]. The abdomen of the artifact is octagonal, with different patterns engraved on each face. The exterior wall of the artifact is entirely covered with a fish-toe circle, while the outer aspect of the ring foot is embellished with an upward lotus motif.

### 2.2. p-XRF

Utilizing the Niton XL3t800 p-XRF (Thermo Fisher Scientific Inc., Waltham, MA, USA) allowed us quickly obtain the elemental composition of the cultural relic’s surface. It is a rapid and precise non-destructive testing method. This instrument adopted high-performance micro-X-ray tubes and high-resolution Si-PIN detectors. The instrument was calibrated based on the basic parameter method and has an automatic calibration function. Before measurement, the equipment status was verified using the calibration block provided by the instrument. To choose a testing position with a smooth surface that fully covered the detection window, the detection mode for the cultural relics was the Precious Alloys mode. The single-eared octagonal gold cup is a national first-class cultural relic and had to follow the principle of non-destructive testing, without any chemical/physical treatment on the cup. The single test time was set to 30 s. To ensure that the data were representative, we selected 5 different substrate positions on the gold cup for measurement. Each point was tested 3 times, and after normalizing the measured data, we calculated the average value. The voltage was 50 kV, the spot diameter was 3 mm, and the data were automatically processed by the instrument’s built-in software and matching Niton Data Transfer (NDT) software.

### 2.3. Ultra-Depth Field Microscope

The 2D or 3D microscopic images of the cultural relic were obtained utilizing a VHX-6000 ultra-depth field microscope (Keyence Corporation, Osaka, Japan). The microscope was equipped with a CMOS image sensor, with effective pixels of 1600 (H) × 1200 (V), and adopted a progressive scanning method, with a maximum frame rate of 50 F/s. A VH-ZST lens was used in imaging. The standard lens provided magnification ranging from 20× to 200×, with a maximum magnification reaching 5000×, and the maximum resolution was 400 nm. During the image acquisition process, the real-time depth-of-field synthesis function was enabled. With just one click, the entire depth-of-field range of the sample was automatically scanned and combined into a full clear image. All images were saved in JPG format. The above imaging conditions and acquisition strategies ensured the clarity, representativeness, and repeatability of the observation results.

## 3. Results

### 3.1. Composition Analysis

**Figure 3:**
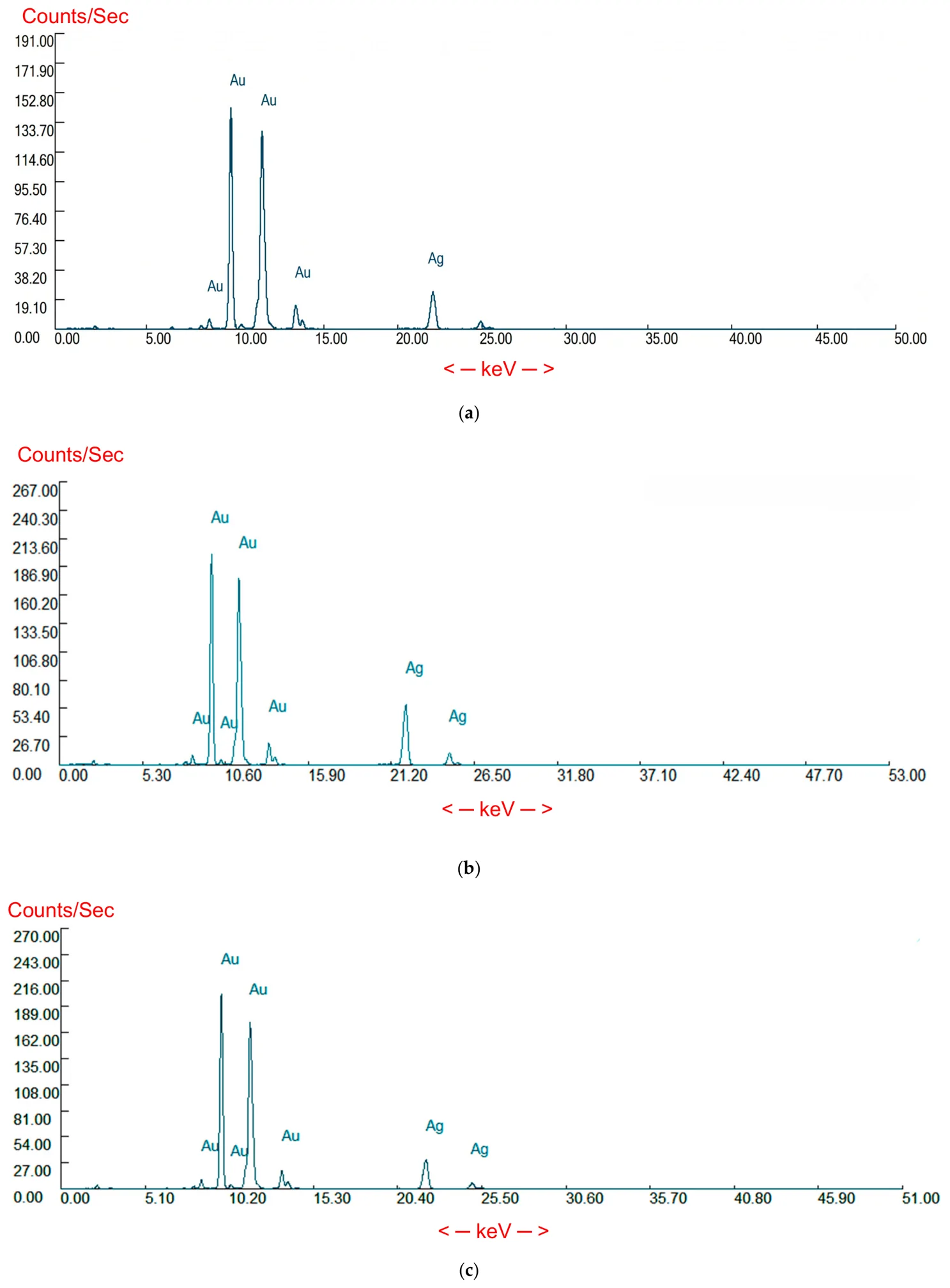
(**a**) The p-XRF spectrum at the exact center of the ring foot. (**b**) The p-XRF spectrum at the pearl roundel on the abdominal ridge. (**c**) The p-XRF spectrum at the pearl roundel on the rim. Note: X-axis (X): keV (kilo-electron volts). This represents the energy of characteristic X-rays. Each element has a fixed characteristic energy peak, which is used for qualitative identification of element types. Y-axis (Y): Counts/Sec (CPS, counts per second). This stands for the intensity of X-ray photons received by the detector. A higher peak corresponds to a higher relative content of the corresponding element.

In this composition analysis, samples were collected from the abdomen pattern of the gold cup, the exact center of the ring foot, and the pearl roundel on the abdomen. The detailed results of the composition analysis are presented in Table 1. The base material composition results show that the material composition of the single-eared octagonal gold cup was similar at different base locations, with gold content ranging from 84% to 88% and silver content ranging from 10% to 13%. Figure 3a–c are the p-XRF spectra at the exact center of the ring foot and the pearl roundel on the abdominal ridge and rim, showing dominant Au with minor Ag content. Therefore, it can be concluded that the material of the gold cup is Au-Ag alloy.

### 3.2. Manufacturing Technology Observation

**Figure 4:**
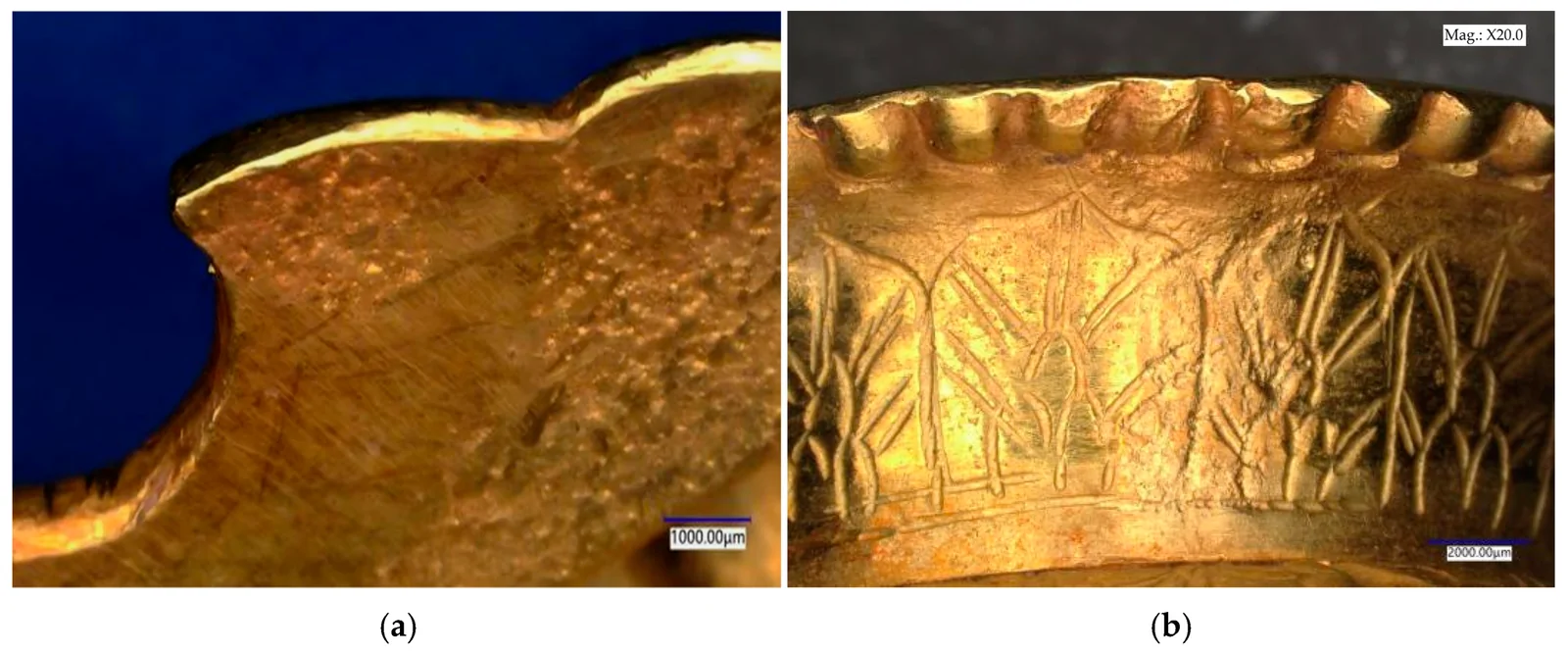

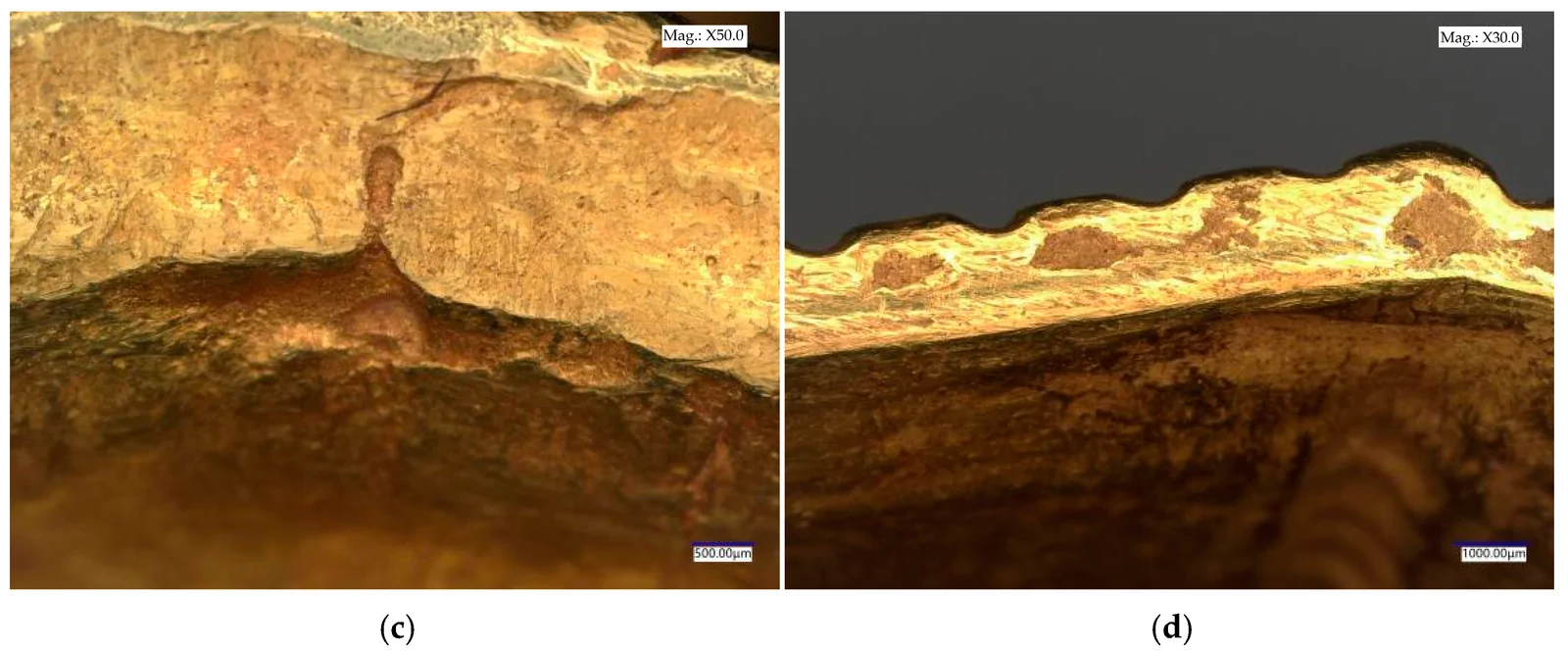
(**a**) Shrinkage cavities in the back of the fish-tail-shaped finger pad. (**b**) Shrinkage cavities in the front position of the ring foot. (**c**) Shrinkage cavities in the interior wall of the ring foot. (**d**) Shrinkage cavities in the interior wall of the rim.

**Figure 5:**
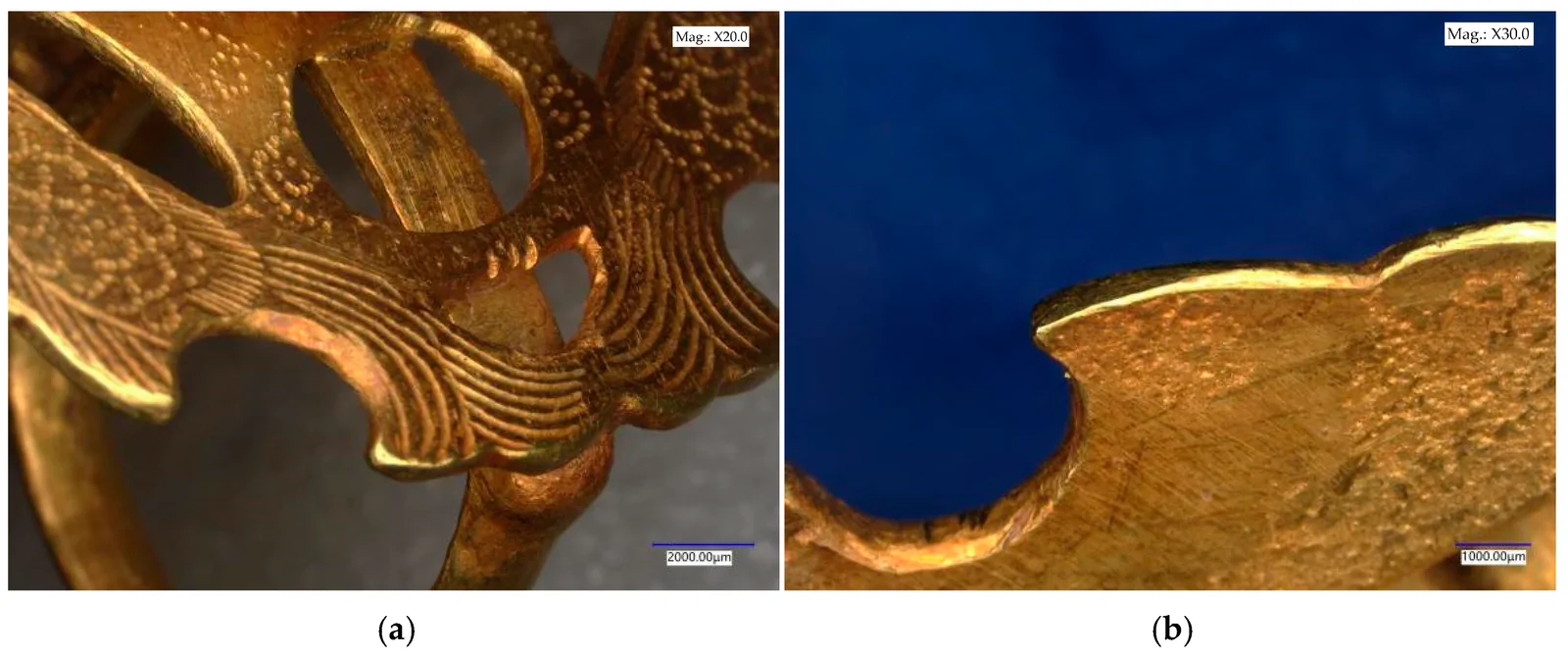
(**a**) Polishing treatment in the front position of the leaf-bud-shaped wing handle. (**b**) Polishing treatment in the back of the fish-tail-shaped finger pad.

The ultra-depth field microscope was used to examine the manufacturing technology of the single-eared octagonal gold cup, with particular focus on observing manufacturing marks and defects on the surface and at the connection parts [23]. Under the ultra-depth field microscope, some shrinkage cavities were observed on the back of the fish-tail-shaped finger pad, the front position of the ring foot, and the interior walls of both the ring foot and the rim (Figure 4a–d). The shape of the shrinkage cavities was irregular, and the inner wall of gold cup was rough. Ref. [23] used an ultra-depth field microscope to observe the rim of the cup body, the body of the wild goose, and the flower leaf of silver cup unearthed from the Weishi family tomb of the Tang Dynasty. It was found that there were some shrinkage cavities in these parts. Although these parts were polished, the shrinkage cavities could still be seen, indicating that the silver cup was cast. The shrinkage cavities at different positions of the gold cup were very obvious. Figure 2 and Figure 4c,d show that the wall of the gold cup is thicker, and the weight of the gold cup was 67.004 g. Through comparison, it was found that the bowl unearthed from the Tomb of State Chen Princess has cracks at the curved position of the bowl. Microscopic observation revealed that the wall of the bowl is relatively thin, and no welding marks were found at the curved position of the bowl. It was inferred that the bowl was made by hammering. In addition, it was inferred from ref. [23] that the two gilded silver boxes were formed using hammering due to the thin walls being around 0.3 mm. In summary, the characteristics of wares formed by hammering are thin walls and smooth surfaces. On the contrary, it was inferred that the single-eared octagonal gold cup was cast, due to its thick wall, heavy weight, irregular shrinkage holes, and rough inner wall. Upon microscopic examination, it was observed that both the front position of the leaf-bud-shaped wing handle and the back of the fish-tail-shaped finger pad had undergone grinding and polishing treatments (Figure 5a,b). It was inferred that the polishing on the back of the fish-tail-shaped finger pad was intended to conceal the marks of shrinkage holes, but these cavities remained conspicuously visible.

**Figure 6:**
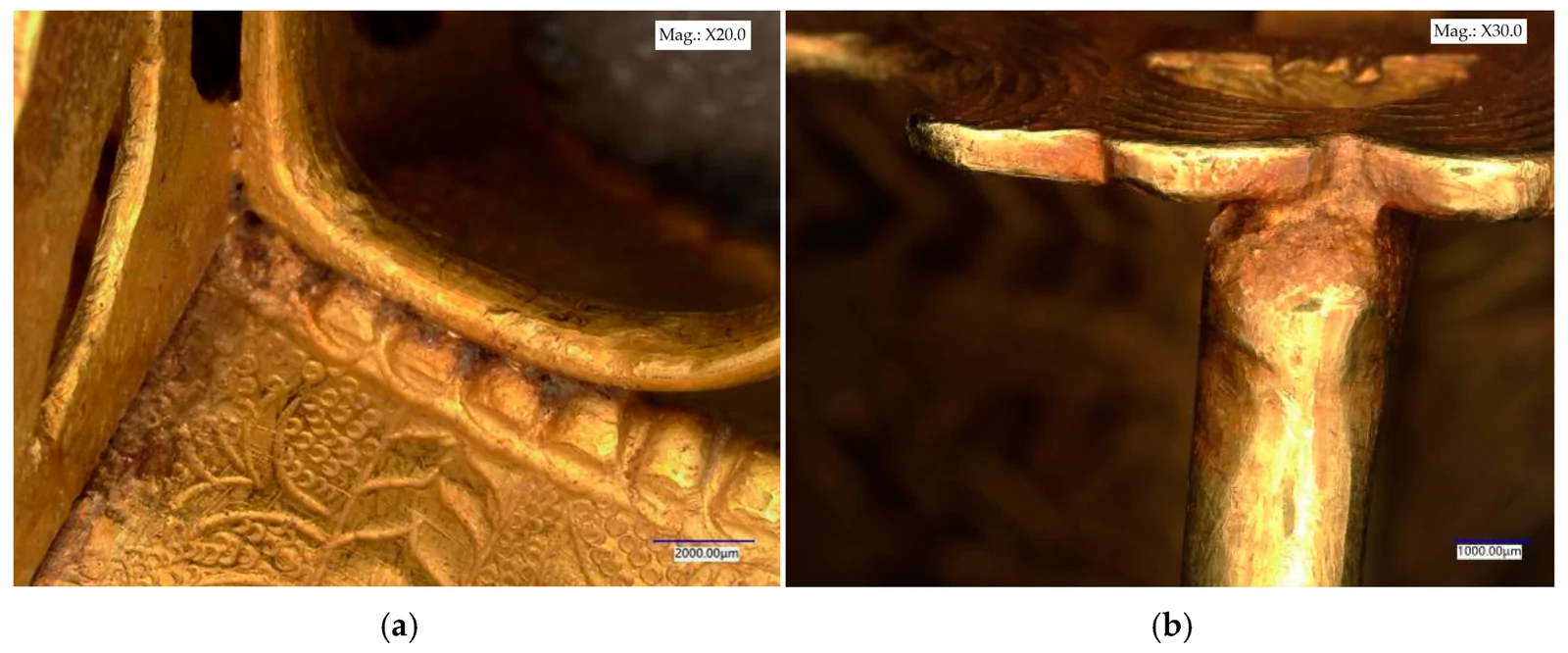
(**a**) Spot and surface welding between the cup body and the wing handle. (**b**) Spot and surface welding between the finger pad and the wing handle.

**Figure 7:**
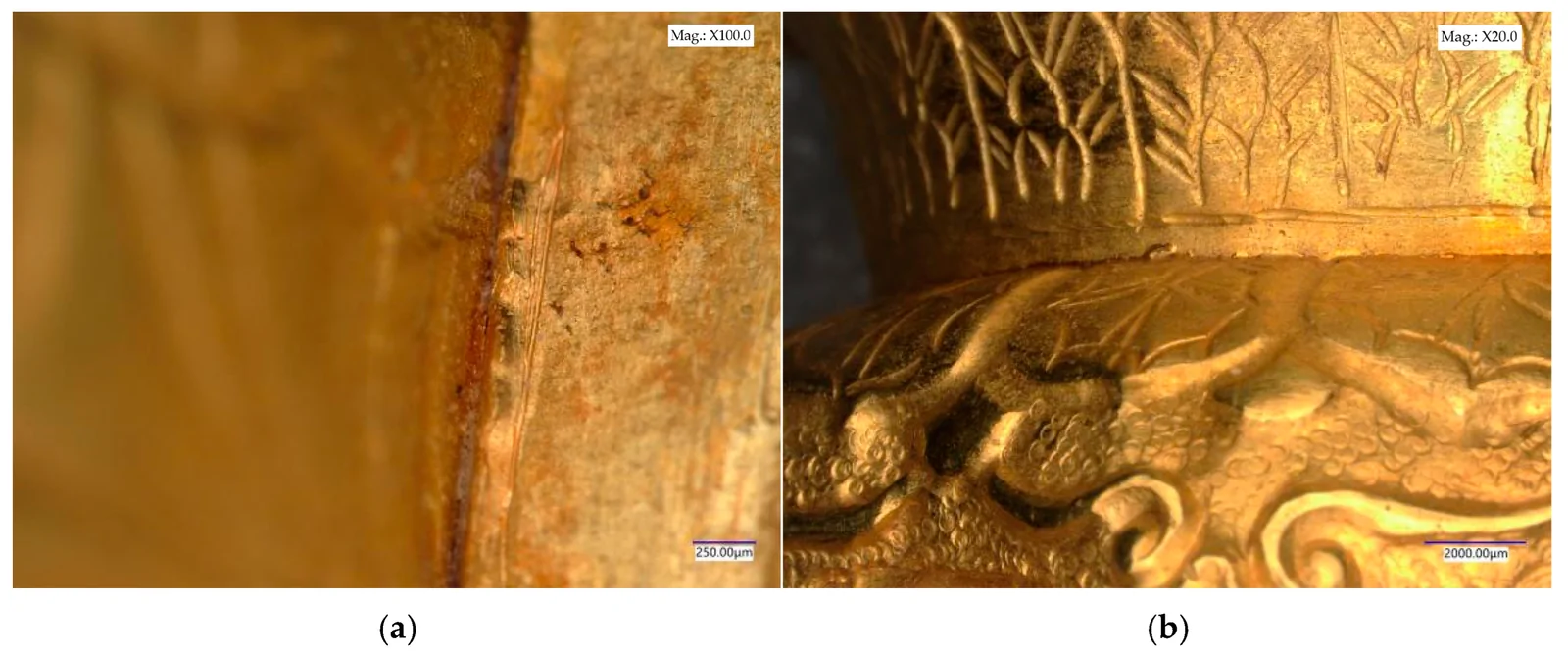
(**a**) The weld seam of the cup body and the ring foot. (**b**) Insufficient melting of the welding material.

Using the ultra-depth field microscope to conduct microscopic observation on the gold cup, it was found that welding technology was employed at all connection positions, including weld seams and welding spots. Specifically, it is likely that there was spot and surface welding between the cup body and the wing handle at one location (Figure 6a), spot and surface welding between the finger pad and the wing handle at one location (Figure 6b), and a continuous weld seam around the entire perimeter between the cup body and the ring foot (Figure 7a). It is worth noting that the welding flux at the joint between the cup body and the finger pad was not completely fused. Indirect gaps could be observed in the weld seam, which might be attributed to the three-dimensional pattern of the pearl roundel. There is green solder between the weld seam of the cup body and the ring foot (Figure 7a). Green solder marks were also left on the welding points of the ewer and the warming bowl unearthed from the Song Dynasty gold and silver caches in Pengzhou, Sichuan Province. The specific locations were on the spout and lid of the ewer and the ears of the warming bowl [24]. It could be seen that the welding flux in the Tuerji Mountain Tomb of the Liao Dynasty is the same color as that of the Song Dynasty caches. According to the testing results of p-XRF material composition, it was determined that the gold content of green solder was 68%, the silver content was 29%, and the copper content reached 3%. By comparison, it was found that the content of silver and copper elements in the green solder position was much higher than that in the matrix. The melting point of gold is 1065 °C. It was found that the addition of silver and copper could lower its melting point [25], suggesting that craftsmen at that time deliberately increased the content of these two metals in order to reduce the melting point of the welding material. According to the phase diagram of the gold–silver–copper ternary alloy [26], the total molar fraction of gold, silver, and copper was complete. In the ternary phase diagram, it was located in the upper half of the Ag-Au-Cu triangle, close to the Au-Ag binary boundary line, and was found to belong to a medium gold–high silver–low copper ratio. Specifically, Au60% corresponded to 900 °C, and Au70% corresponded to 950 °C. The melting point of the welding flux was determined to lie between the 900 °C and 950 °C isotherms. It could be seen that during the welding process, due to the low welding temperature, the welding material was not completely melted, resulting in green solder marks. In addition, insufficient melting of the welding material was observed at the weld seam connecting the cup body and the ring foot (Figure 7b). This issue might be due to the craftsman’s positional deviation when placing the welding material or failure to control the temperature required for melting materials [27].

**Figure 8:**
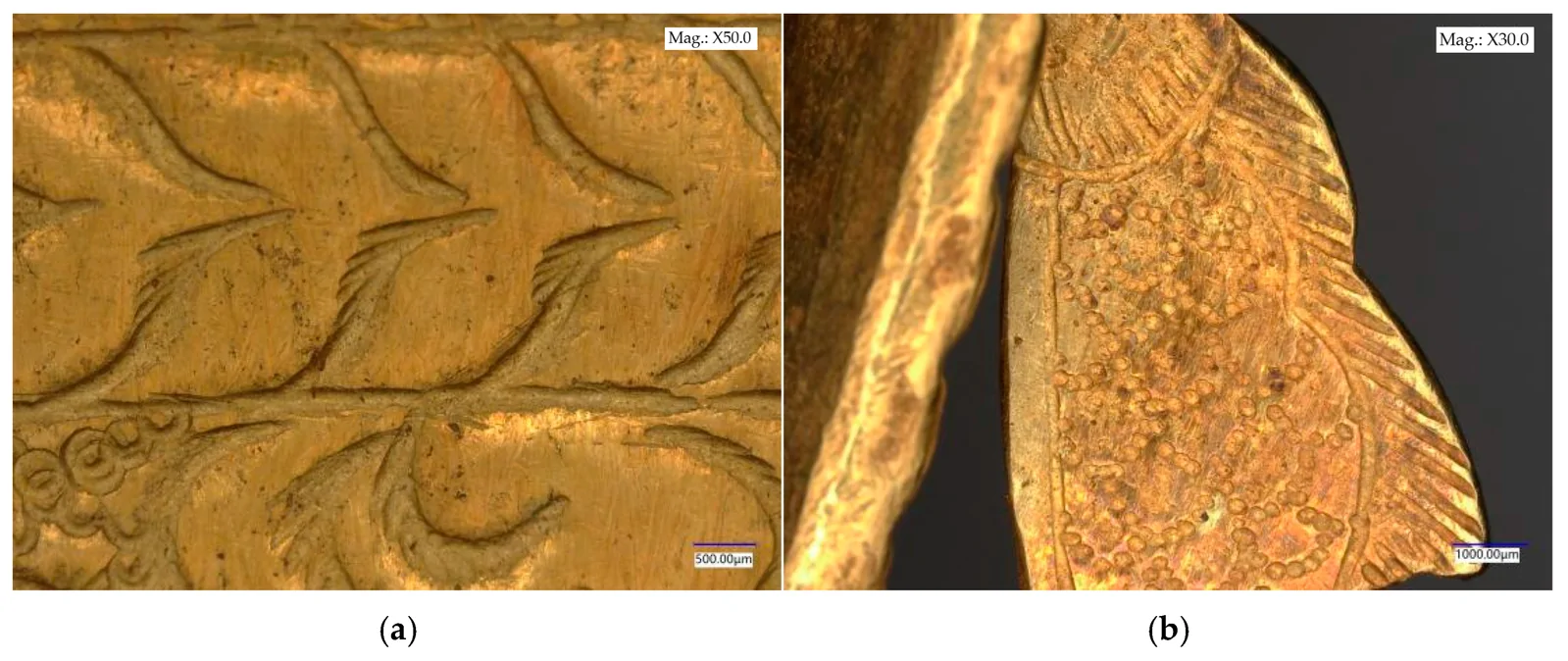
(**a**) Straight-headed graver in abdomen patterns. (**b**) Point-headed graver and straight-headed graver in the fish pattern.

**Figure 9:**
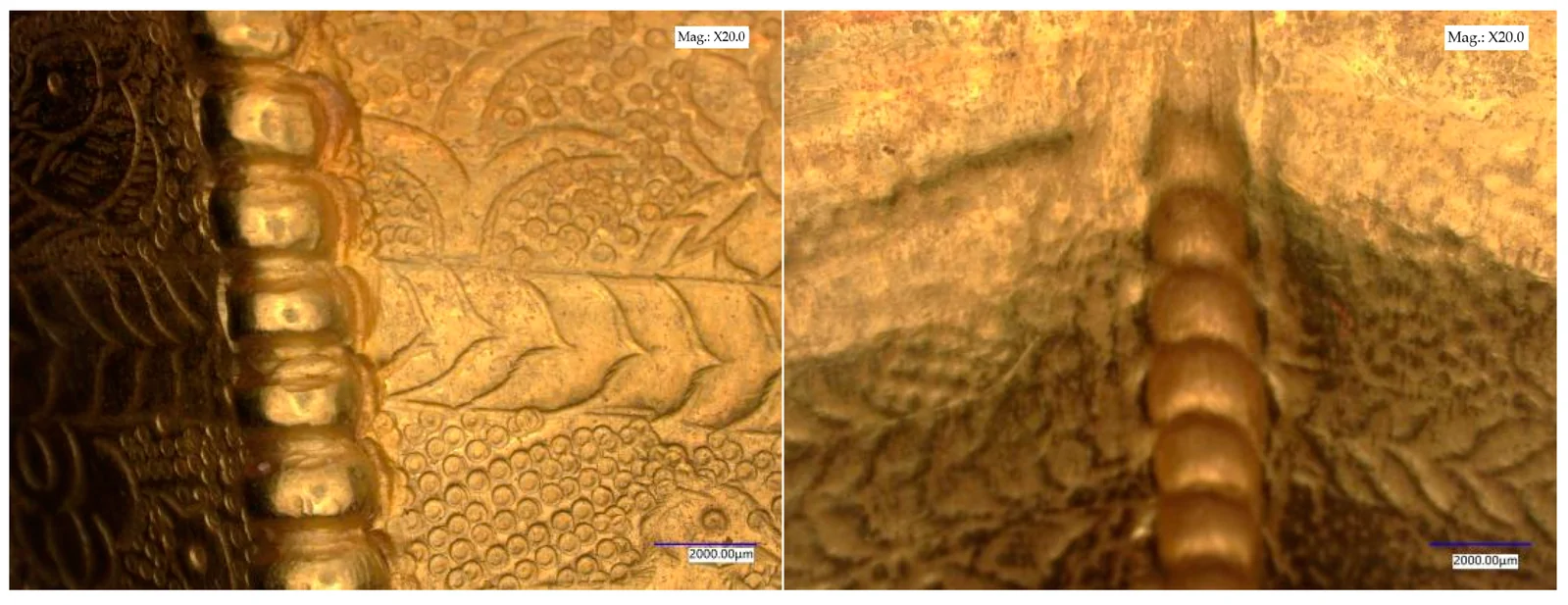
The three-dimensional pearl roundel on the front and back sides of the eight edges.

**Figure 10:**
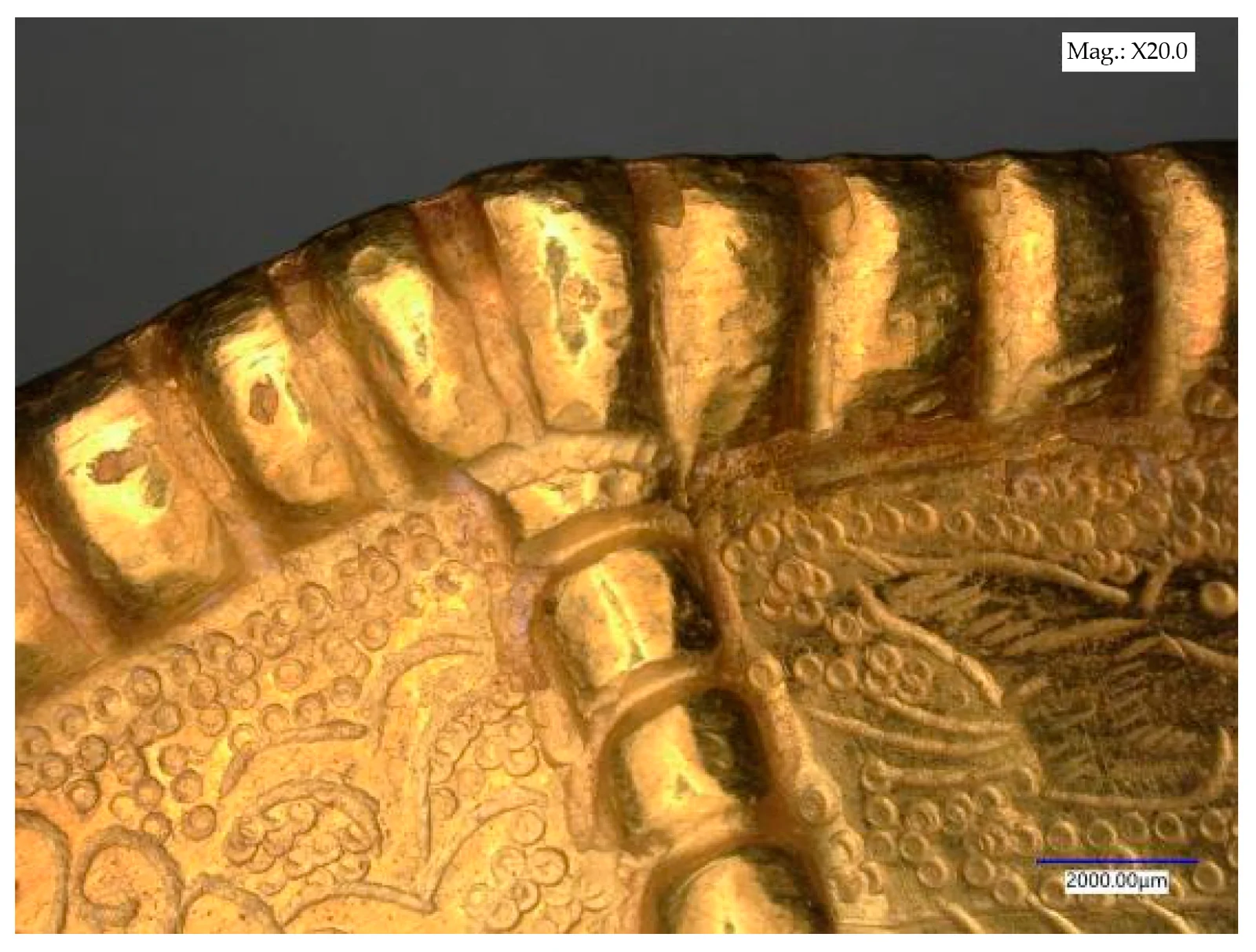
The three-dimensional pearl roundel in the rim.

**Figure 11:**
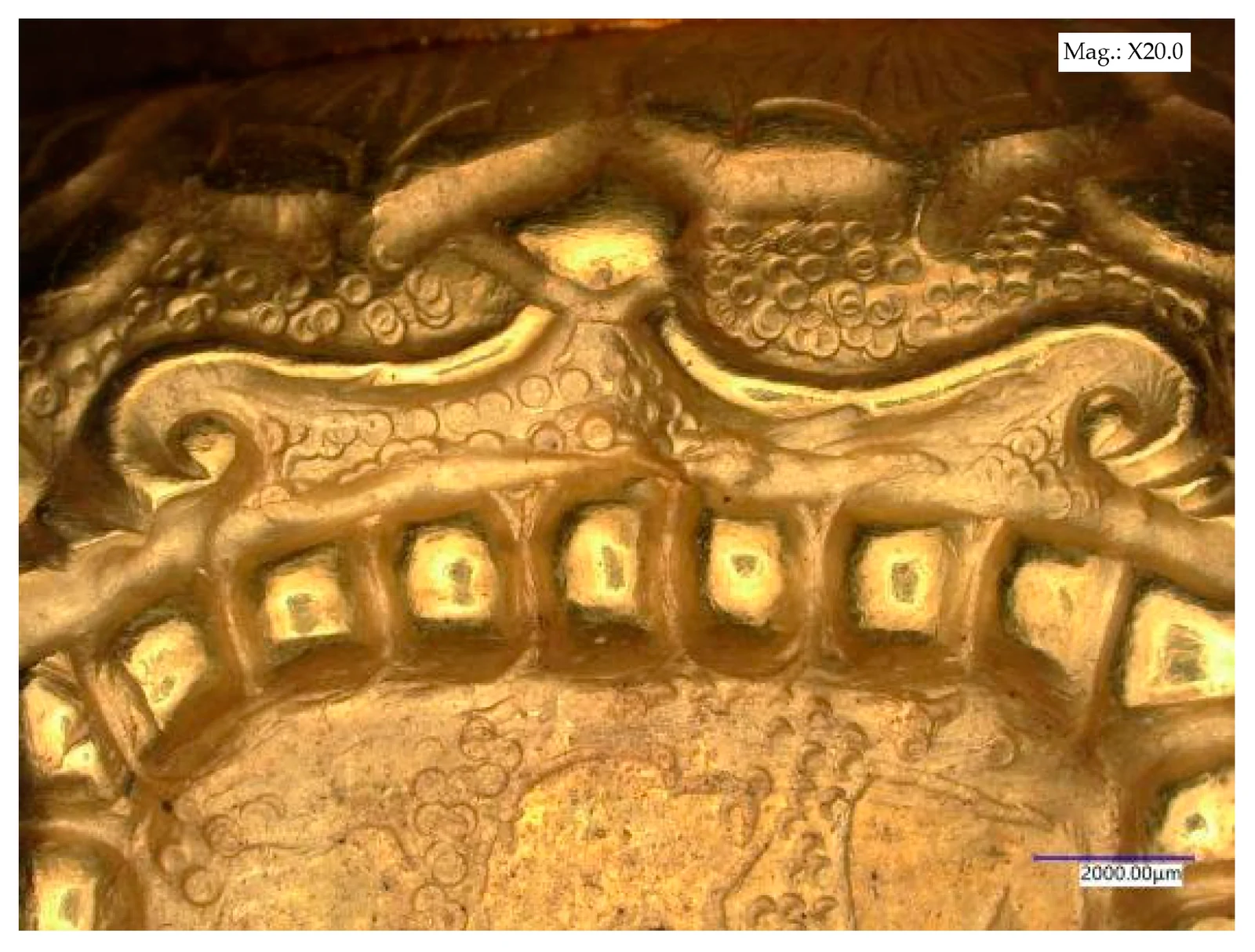
The three-dimensional upward lotus motif in the cup body bottom.

Assessing engraving technology mainly involves observing the shape, continuity, similarity, and defects in the artifact’s pattern lines, followed by an inference about the tools used during the manufacturing process [23]. The manufacturing marks on the surface patterns of the gold cup were observed using an ultra-depth field microscope. The detailed observation results are shown in Table 2. The outside of the gold cup has eight abdomen surfaces, all of which are located in different planes. Microscopic observation preliminarily revealed that the abdomen patterns are composed of overlapping lines made with a straight-headed graver one by one (Figure 8a). The fish-toe circle on the abdomen was fully engraved with a bead-carving graver, featuring concave edges. The fish-toe circle overlapped in some locations, with equal diameters, and the average diameter of the fish-toe circle was 303 μm. The fish-toe circle diameter on the Tang Dynasty gilded silver box unearthed from the Xiaoliuzhuang Tomb was 200 micrometers [20], while the average diameter of the fish-toe circle on the chased gold needle case unearthed from the Tomb of Princess Chen of the Liao Dynasty was 600 μm [27]. It was observed that the chisels employed for the fish-toe circle in the Tang and Liao dynasties were similar. In response to the different types of objects and the requirements of the overall decorative design, craftsmen utilized engraving implements of varying specifications. It was tentatively observed through microscopy that the fish pattern on the fish-tail-shaped finger pad was formed by using a point-headed graver to create the scales and eyes, while the line pattern of the fish body was made with a straight-headed graver (Figure 8b). There was a draft line beneath the trellis-patterned rosettes outside the ring foot. The three-dimensional pearl roundels on the front and back sides of the eight edges (Figure 9), the rim (Figure 10), and the ring foot of the gold cup are on different planes. Just like the three-dimensional upward lotus motif (Figure 11), the technique of a three-dimensional graver was employed to carve out the edges of the desired pattern from the back of the artifact. The depth and height of the engraving were determined based on the pattern area, presenting a three-dimensional effect. The distance between the two connected pearl roundels on the abdominal ridge was measured as 745.3 μm.

## 4. Discussion

### 4.1. Material and Manufacturing Technology Analysis

Here, p-XRF was used for non-destructive analysis on the abdomen, ring foot, pearl roundel on the ridges, and pearl roundel on the rim of the single-eared octagonal gold cup. The composition analysis results, as presented in Table 1, revealed that the elemental content ratios remained consistent at various locations of the cup base. This suggested that the craftsman may have deliberately alloyed the obtained natural gold raw material.

Casting technology has a long history in China, and it had already been the main method for shaping gold and silver artifacts before the Warring States period. Casting technology is a manufacturing technology in which metal is melted at high temperatures and then poured into a mold. After it cools and solidifies, the desired shape of the artifact is finally formed. The casting technology involves melting gold and silver artifacts into a liquid state and casting it with a mold, which is similar to the manufacturing method of Chinese bronze, except for the raw materials [28]. Through observation with an ultra-depth field microscope, shrinkage cavities were identified on the finger pad, the ring foot, the inner wall, the abdomen, and the rim of the single-eared octagonal gold cup, preliminarily suggesting that the gold cup was molded using casting technology.

The gold cup unearthed from the tomb of Marquis Yi of Zeng was made into separate parts, such as the cup body, base, and handle, by casting technology and then connected as a whole using welding technology [29]. The gold cup in this article used the same casting and welding techniques as the gold bowl unearthed from the tomb of Marquis Yi of Zeng. Based on the preliminary judgment of the manufacturing techniques of the four silver wares unearthed from the Wei family tomb [23], through the microscopic observation results, a preliminary judgment could be made on the manufacturing technology of the single-eared octagonal gold cup unearthed from the Turki Mountain Tomb as follows: The cup body of the single-eared octagonal gold cup employed a mold to achieve its specific shape. After the cup body and the ring foot were manufactured separately using casting technology, the ring foot was welded onto the bottom of the cup body. The finger pad and the wing handle were joined together through welding, and then the wing handle was welded onto the cup body. Upon microscopic observation, it was preliminarily discovered that the craftsman took the bottom of the ring foot as the draft line, and the main pattern was engraved on the gold cup surface as the draft. Finally, on the basis of the engraved pattern, the main pattern and the fish-toe circle were engraved. The gold cup made via the casting technique exhibited a three-dimensional craftsmanship effect, and the various accessories produced by casting required welding to be joined to the cup body.

**Figure 12:**
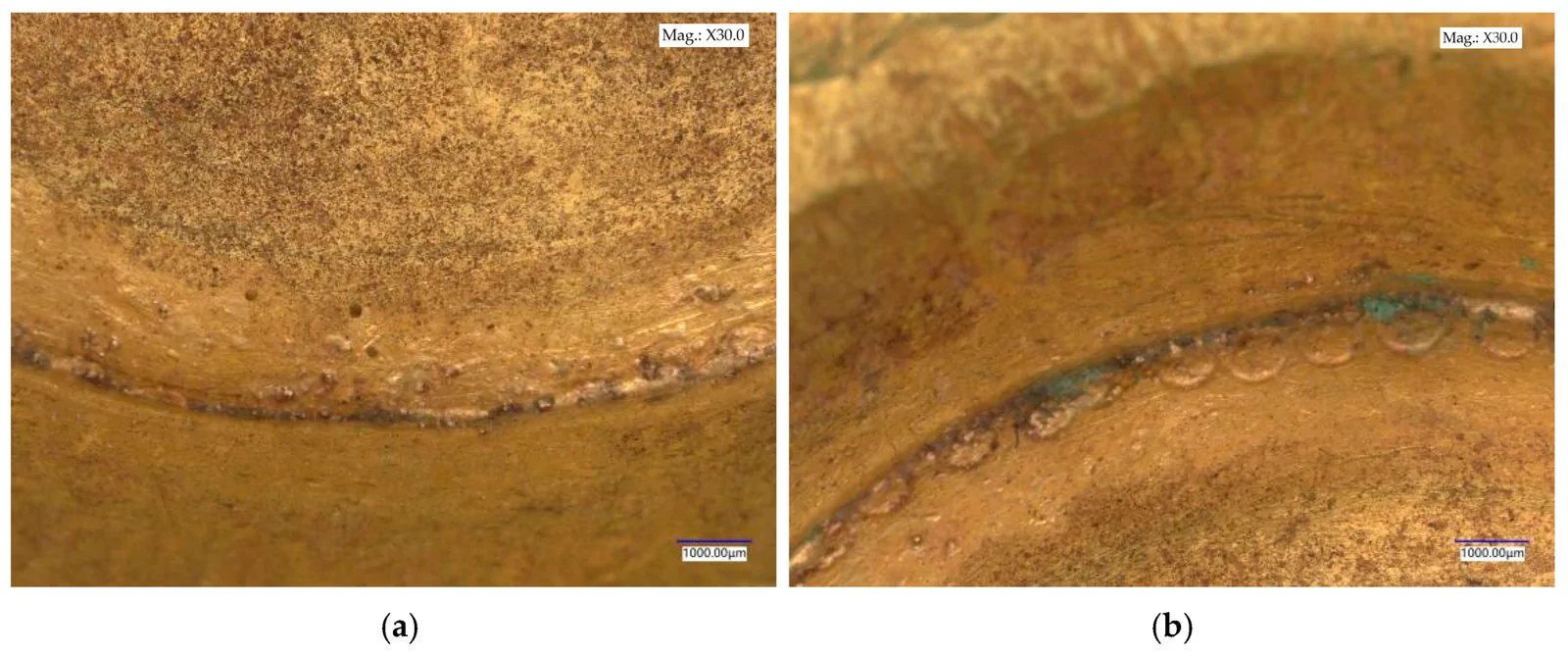
(**a**) The welding small pores in the inner wall of the ring foot. (**b**) Fish-toe circle engraved at the welding position.

Based on the size of the welding area, it could be divided into line-to-line welding and point-to-surface welding [30]. The gold mask unearthed from the Zhaosu Tomb in Xinjiang had its welding position located in the middle of the face, which is a typical example of line-to-line welding [30]. Similarly, the single-eared octagonal gold cup also belongs to the category of line-to-line welding, featuring a small contact area and fine lines. The cup body and ring foot were welded together by precisely aligning their edges using line-to-line welding. Incomplete melting of the welding material was observed at the welding position between the cup body and the ring foot (Figure 7b). In comparison, the welding areas between the cup body and finger pad, as well as between the finger pad and wing handle, were large and did not exhibit the characteristic of fine lines. Therefore, it was preliminarily judged and inferred that these two welding methods belonged to point-to-surface welding. Specifically, it was initially considered that the cup body and finger pad were welded by precisely aligning pearl roundels with the wing handle and joining them using a point-to-surface welding method. We preliminarily judged that the fish-tail-shaped finger pad and leaf-bud-shaped wing handle were welded by precisely aligning the finger pad with the wing handle and joining them using a point-to-surface welding approach. Notably, apart from the small pores observed at the welding position on the inner wall of the ring foot (Figure 12a), the fish-toe circle was also engraved at the same position (Figure 12b). We preliminarily judged that the purpose of decorating this position with patterns was to conceal the welding marks and achieve an aesthetic effect. In addition, red residue was found at the ring foot of the gold cup, and its specific composition required further analysis using micro-Raman spectroscopy.

The engraving technology mainly utilizes a graver as the primary tool and requires a perfect coordination between the graver and a hammer to engrave out exquisite patterns. The surface patterns of the single-eared octagonal gold cup include various patterns, such as a woman carrying a child pattern, twin antelopes pattern, infant playing pattern, twin deer pattern, three people talking pattern, twin elephants pattern, dressing pattern, upward lotus motif, three-dimensional pearl roundel, trellis-patterned rosette, and fish-toe circle. The decorative patterns on the gold cup include figural narrative scenes, animal patterns, and plant patterns. The gold cup was a daily drinking vessel burial object. The pictures of a woman carrying a child, the infant playing, the three people talking, and the dressing depicted daily life and social scenes, expressing the belief that the tomb owner brought the prosperous and comfortable life of his earthly existence into the afterlife. Deer and sheep were traditional Chinese decorative patterns, featuring the characteristics of grassland culture [31]. The Khitan people were a nation on horseback, moving from place to place in search of water and pasture. These two patterns reflected the lifestyle of the nomadic people of the Liao Dynasty who engaged in animal husbandry. Deer were beloved auspicious beasts and played an important role in the hunting life of the Khitan people. The tomb owner was a shaman [32]. The animal patterns might be related to the shamanic belief in animal spirits or the concept of spiritual assistance, serving as a medium for communication between heaven and earth. The floral medallion pattern was concentrated in the early and mid-early Liao Dynasty and was the main feature of this period [2]. As symbols of sanctity, solemnity, and wealth in Buddhism, an upward lotus motif and trellis-patterned rosette embodied the noble identity of the tomb owner and their spiritual pursuit of the afterlife. The extensive use of gold wares among the nobility of the Liao Dynasty had a long history, which was closely related to the idea dating back to the Han Dynasty that using gold and silver tableware could prolong life, achieve immortality, and ascend to heaven. When the gold cup was buried, its function changed from practical tableware used in life to a ritual vessel after death. The patterns on the gold cup were influenced by the noble concept of treating the dead as if they were still alive in the Liao Dynasty, integrating Buddhist and Shamanic ideas. It was a materialized expression of cultural integration and the shamanic identity of the tomb owner.

**Figure 13:**
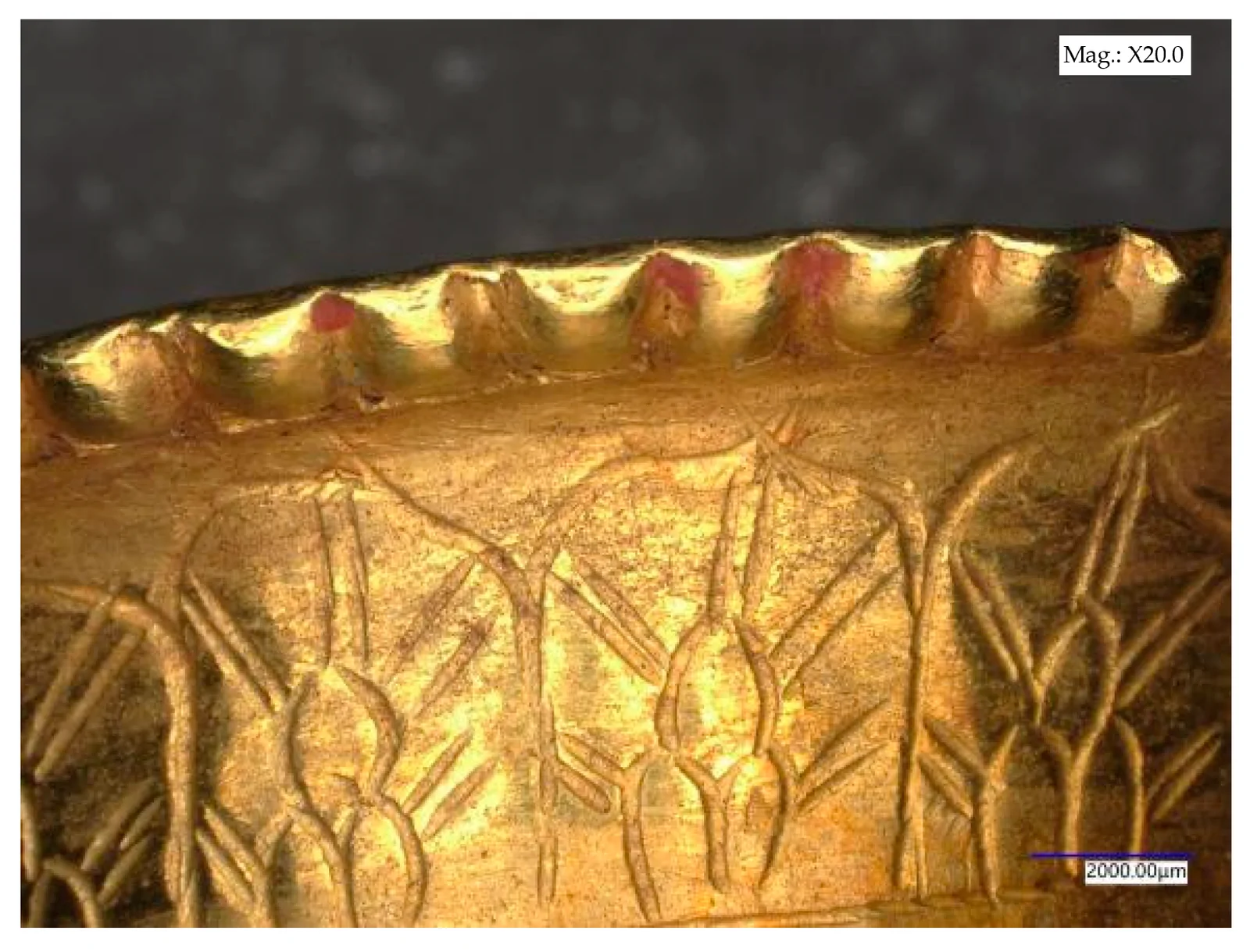
The draft line in the ring foot.

In addition, a draft line was discovered at the bottom of the gold cup’s ring foot (Figure 13). The discovery of the draft line is not unique to the Liao Dynasty. Gold ornamentals unearthed from the Xigou site in Balikun, Hami, Xinjiang (late Warring States period to the early Western Han Dynasty) [33,34], and the silver cup from the Chang’an Wei family tomb (Tang Dynasty) [23] both revealed the presence of draft lines. This observation suggested that the draft line on the single-eared octagonal gold cup to some extent inherited the manufacturing technology from the late Warring States period to the early Western Han Dynasty and the Tang Dynasty. Ancient craftsman used tools to engrave out a rough outline on the surface of the gold cup as a draft and then officially drew the patterns based on the draft.

**Figure 14:**
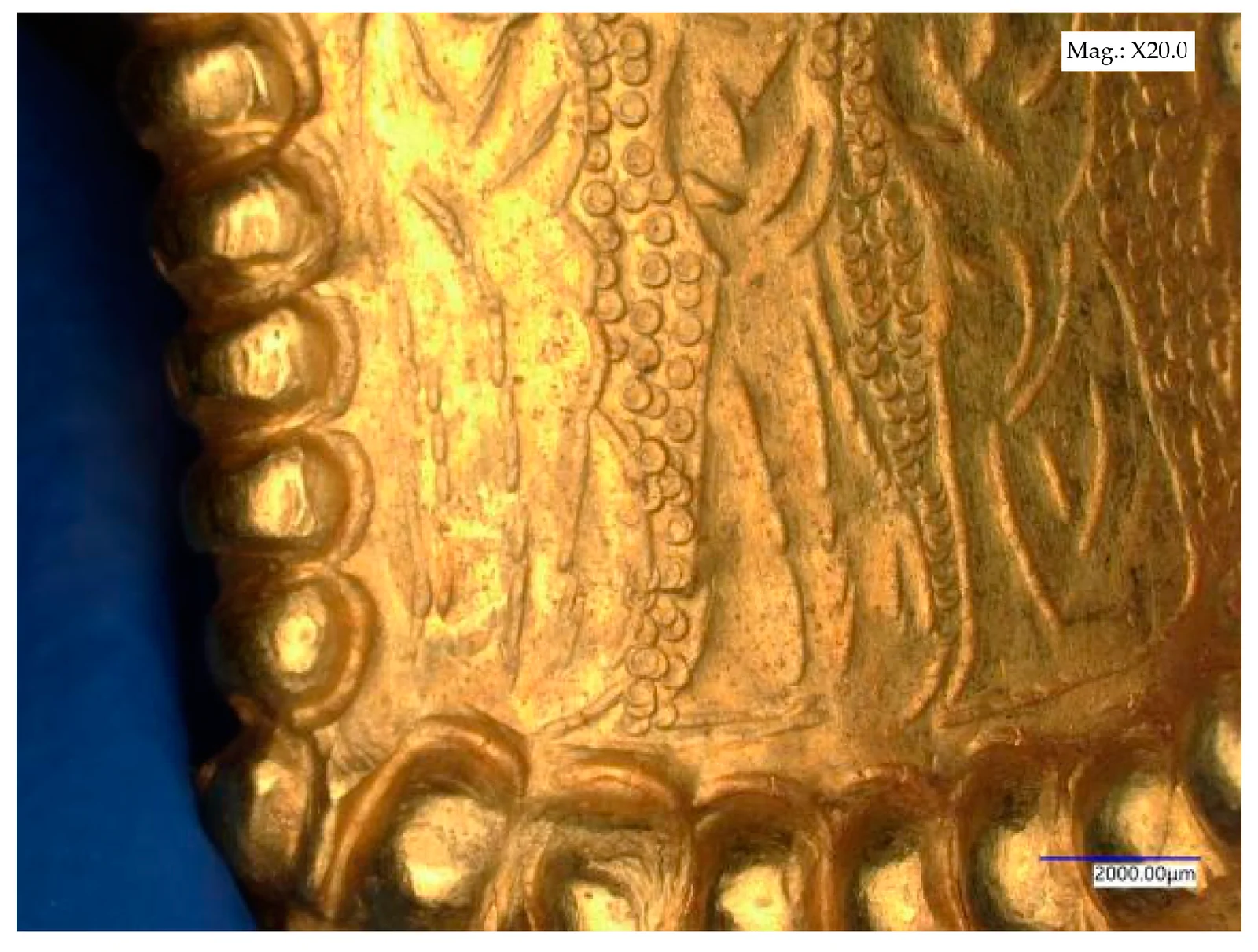
The overlapping marks of the patterns.

The single-eared octagonal gold cup was engraved with different engraving tools to create patterns. In the book of Chinese Fine Gold Manufacturing Technology and Cultural Relics [35], there are detailed names for the graver heads. In this paper, microscopic observation preliminarily revealed that the manufacturing of gold cup primarily utilized a straight-headed graver, bead-carving graver, three-dimensional graver, and point-headed graver. The abdomen patterns were composed of overlapping lines made with straight-headed graver, and it could be inferred that the engraving direction of the straight-headed graver was generally from left to right. The cross-section of the point-headed graver was a concave hemisphere, and the fish pattern on the fish-tail-shaped finger pad was likely to be created using a point-headed graver to form the fish scales and eyes (Figure 8b). The cross-section of the fish-toe circle graver head was a concave hemisphere. The average diameter of the fish-toe circle measured was 303 μm, suggesting that the craftsman employed a bead-carving graver to craft the fine and dense circles. Microscopic observation revealed that the overlapping marks of the patterns were evident (Figure 14). It could be observed that fish-toe circle was interrupted and covered by the flower patterns under a 50× objective lens in Figure 8a. The covering details observed in Figure 14 further illustrated the sequence of making the pattern. Under a 20× objective lens, it was also observed that fish-toe circle was interrupted and overlaid by the flower patterns. Both Figure 8a and Figure 14 directly observed the engraving sequence of the gold cup. It is speculated that the engraving sequence was initially engraving the fish-toe circle, followed by the engraving flower patterns on the cup’s surface, indicating that the craftsman did not execute the engraving in a single step. Ref. [20] found through microscopic observation that the fish-toe circle was interrupted and covered by the flower patterns. The author believed that the engraving sequence was to first engrave the fish-toe circle and then the flower patterns. In the literature [27], through microscopic observation, it was found that the chiseling marks had damaged the fish-toe circle, and the overlapping marks of engraving were obvious. Judging from the overlapping marks of the patterns, the sequence of the artisans’ engraving was to first engrave the fish-toe circle and then the intertwining vine pattern. By comparison, it was found that the sequence of engraving patterns on the artifacts of the Tang and Liao Dynasties was consistent. The fish-toe circle was engraved first, followed by the other patterns. In summary, this demonstrates that the craftsman designed the overall engraving sequence according to the requirements of the artifact pattern. In ancient times, when crafting a gold cup without the aid of a magnifying glass, a craftsman relied solely on their experience to engrave patterns, which inevitably resulted in overlapping patterns.

### 4.2. Methodological Limitations and Preliminary Nature of Interpretations

This study mainly relied on two non-destructive techniques: p-XRF and an ultra-depth field microscope 3D microscopic system. Although they were suitable for rapid on-site screening and surface morphology observation, their limitations have to be acknowledged. First, p-XRF is a surface analysis method with limited penetration depth (typically <100 μm), and its analytical results were significantly affected by matrix effects, surface moisture, particle size, and sample homogeneity. Consequently, its quantitative accuracy was inferior to that of laboratory wavelength-dispersive XRF; the obtained element concentrations were only semi-quantitative, with insufficient detection sensitivity for light elements, and thus it could not replace the precise quantification achieved by wet chemical methods or ICP-MS.

The ultra-depth-of-field microscope could provide high-precision 2D/3D micro-area topography and chromatic information, but it was limited to optical morphology and could not acquire elemental or phase composition. Moreover, constrained by the optical diffraction limit, it could not achieve nanoscale lattice resolution, and it also lacked the capability for simultaneous coupled analysis of in situ chemical composition or crystal structure. Furthermore, constrained by the preciousness of the samples and the requirement for non-destructive analysis, this study did not carry out supplementary analyses such as SEM-EDS, XRD, or LA-ICP-MS. Therefore, our interpretations of the correlations between elemental distributions and microstructural features, as well as our inferences concerning surface alteration processes and the manufacturing technology traces, were all preliminary and needed to be verified by subsequent higher-resolution micro-area analytical techniques.

### 4.3. Expanded Discussion of Surface Alteration Effects on p-XRF Analysis

The single-eared octagonal gold cup unearthed from the Turki Mountain Tomb of the Liao Dynasty, like many archaeological gold objects, underwent various surface alteration processes during its long burial history. These processes—including corrosion, patination, and surface enrichment—could significantly affect the results of portable X-ray fluorescence (p-XRF) analysis, which is inherently a surface-sensitive technique. Below, we discuss the three primary alteration phenomena and their implications for our compositional interpretations.

Gold alloys buried in archaeological contexts are subject to corrosion driven by prolonged exposure to soil moisture, dissolved salts, and varying redox conditions. Although gold itself is chemically noble and does not readily corrode, the alloying elements—particularly copper and silver—are susceptible to corrosion. On the surface of the cup, copper might oxidize to form cuprite (Cu_2_O) or other copper corrosion products, while silver might form silver chlorides or sulfides. These corrosion products manifested as patination layers that could be visually observed as discoloration or tarnish patches. The critical issue for p-XRF analysis was that the corroded surface layers still contributed to the total X-ray signal measured, even though the beam penetrated deeper into the core. This meant that the p-XRF spectrum represented a composite signal from both the altered surface layer and the underlying bulk metal, with the relative contribution of each depending on the thickness and composition of the corrosion layer, as well as the excitation energy of the X-ray source. As a result, quantitative analysis performed under the assumption of a homogeneous sample might yield compositions that were systematically biased.

Long-term soil diagenesis precipitated thin patina layers of iron oxides, copper carbonates, and platinum-bearing mineral precipitates onto the cup’s exterior. These heterogeneous secondary crusts directly contributed exogenous Fe, Cu, and Pt signals to p-XRF spectra. The trace Pt detected in our measurements likely originated in part from burial patina contamination rather than intentional alloy addition during Liao metalworking. Patina heterogeneities across the cup’s abdomen and ring foot also created random analytical scatter between replicate measurement points. Selective leaching of less noble Cu/Fe drove a well-documented surface passivation effect termed gold/silver enrichment. The depleted outer surface became relatively concentrated in Au and Ag, artificially elevating their measured wt% values. For instance, replicate p-XRF spots with marginally higher Au (~88 wt%) and lower Cu/Fe reflected stronger surface enrichment, while spots with slightly reduced Au (~84 wt%) corresponded to thinner patina and less severe element leaching. This enrichment effect was the primary driver of compositional variability observed across our five measurement datasets.

This expanded discussion clarifies the limitations of surface p-XRF analysis on archaeologically altered gold wares, strengthens the robustness of our alloy compositional arguments for the Turki Mountain Tomb gold cup, and aligns our analytical interpretation with established archaeological XRF metallurgy standards for buried precious metal artifacts.

## 5. Conclusions

The single-eared octagonal gold cup unearthed from the Turki Mountain Tomb, one of the three most representative Liao Dynasty tombs, showcases the era’s highest level of gold and silver manufacturing technology. Gold possesses the physical property of immortality. When a gold cup was buried in a tomb, its function o shifted from a practical dining vessel used in daily life to a sacrificial utensil after death, embodying the concept of treating death as alive and further strengthening its attribute as a sacred object. The abdomen and ring foot of the gold cup were engraved with animal patterns, plant patterns, and human stories. Considering that the tomb owner was a shaman, these patterns might be scenes of the tomb owner presiding over the priestly and shamanic ceremonies. In this paper, p-XRF and ultra-depth field microscopy were used to conduct composition analysis and microscopic observation on a single-eared octagonal gold cup, and the findings were as follows:

(1) By testing the components of the abdomen pattern, the exact center of the ring foot, the pearl roundel on the abdomen, and the rim, it was found that the component testing results at different base locations were similar, with stable element content ratios. The p-XRF spectra at different positions showed dominant Au with minor Ag content; the gold content ranged from 84% to 88%, and the silver content ranged from 10.1% to 13%, indicating that the gold cup was made of Au-Ag alloy.
(2) Through microscopic observation, it was found that there were some shrinkage cavities on the back of the fish-tail-shaped finger pad, the front position and the inner wall of the ring foot, and the inner wall of the rim; it was inferred that the gold cup was manufactured using casting technology.
(3) The welding technology of the gold cup employed two methods, including line-to-line welding and point-to-surface welding. Green solder and insufficient fusion of welding material were found between the weld seam of the cup body and the ring foot. Based on the analysis of the ternary alloy phase diagram of Ag-Au-Cu, due to the low welding temperature, the welding material was not completely melted, resulting in green solder. In addition, it was inferred that that fish-toe circle was engraved over the small pores on the inner wall of the ring foot to hide them, attaining the aesthetic outcome.
(4) Microscopic observation preliminarily revealed that the manufacturing of the gold cup primarily utilized four types of engraving tools, including a straight-headed graver, bead-carving graver, point-headed graver, and three-dimensional graver. A draft line was initially identified at the ring foot of the gold cup, and it is inferred that the craftsman engraved the draft on the gold cup surface prior to engraving the flower patterns. There were distinct overlapping marks between the fish-toe circle and the flower patterns. After judging, it may have been that the engraving sequence was to engrave the fish-toe circle first, followed by the flower patterns. The pearl roundel and the upward lotus motif were all manufactured using a three-dimensional graver, creating a defined height and depth, thus presenting a three-dimensional pattern effect.
(5) In summary, our preliminary results revealed the composition and manufacturing technology of the single-eared octagonal gold cup, although with the inherent limitations of p-XRF and ultra-depth field microscopy, which included limited detection depth, the inability to test trace elements in deep layers, a lack of lattice and phase structure information, as well as the influence of surface corrosion layers that affected component test results. In the future, these interpretations will require joint validation by complementary techniques.

## Figures and Tables

**Table 1:** Portable X-ray fluorescence spectroscopy (p-XRF) analysis results of the base composition of the single-eared octagonal gold cup unearthed from the Turki Mountain Tomb of the Liao Dynasty.

Serial Number	Detection Location	Average Composition of Alloying Elements (wt%)					Materials
		Au	Ag	Cu	Fe	Pt	
1	The abdomen pattern (three-person standing depiction)	88 ± 1	10 ± 1	1 ± 0.5	0 ± 0.5	1 ± 0.5	Au-Ag
2	The abdomen pattern (dual deer depiction)	88 ± 1	10 ± 1	1 ± 0.5	0 ± 0.5	1 ± 0.5	Au-Ag
3	The exact center of the ring foot	84 ± 1	13 ± 1	1 ± 0.5	1 ± 0.5	1 ± 0.5	Au-Ag
4	The pearl roundel on the abdominal ridge	87 ± 1	12 ± 1	1 ± 0.5	0 ± 0.5	1 ± 0.5	Au-Ag
5	The pearl roundel on the rim	88 ± 1	10 ± 1	1 ± 0.5	0 ± 0.5	1 ± 0.5	Au-Ag

Note: Major and trace elemental compositions of gold alloy samples measured by portable XRF (mass fraction, wt%). All decimal fractions of raw instrumental readings are rounded to integer values in accordance with the measurement credibility of portable XRF. U denotes expanded measurement uncertainty with coverage factor k = 2; U = ±1 wt% for Au and Ag (major components), and U = ±0.5 wt% for Cu, Fe, and Pt (trace components). Uncertainty values are derived from repeated standard reference material measurements under identical analytical conditions.

**Table 2:** Microscopic observation results of engraving technology for surface pattern on the single-eared octagonal gold cup.

Artifact Name	Surface Pattern	Observation Result of Pattern	Engraving Tools
Single-eared octagonal gold cup	1. Eight abdomen patterns 2. Fish pattern (fish-tail-shaped finger pad)	The abdomen patterns were composed of overlapping lines using straight-headed graver one by one. The fish pattern on the fish-tail-shaped finger pad was formed by using a point-headed graver to create the scales and eyes, while the fish body was made by using a straight-headed graver.	Straight-headed graver Point-headed graver
	3. Pearl roundel 4. Upward lotus motif	The inner wall of the artifact was pushed out with a three-dimensional graver to form a three-dimensional pattern.	Three-dimensional graver
	5. Fish-toe circle	The entire abdomen of the gold cup was fully engraved with a fish-toe circle, with concave edges.	Bead-carving graver

## Data Availability

The original contributions presented in this study are included in the article. Further inquiries can be directed to the corresponding authors.
