# Correction: Zhang et al. Research on the Composition and Manufacturing Technology of the Single-Eared Octagonal Gold Cup Unearthed from the Turki Mountain Tomb in Inner Mongolia. *Materials* 2026, *19*, 3082

**DOI:** 10.3390/ma19194134

**Published:** 2026-09-28

**Authors:** Yawei Zhang, Lijuan Dong, Weidong Hu, Lei Yang, Li Li

**Affiliations:** 1 School of Yungang Ology, Shanxi Province Key Laboratory of Microstructure Electromagnetic Functional Materials, Institute of Solid State Physics, Shanxi Datong University, Datong 037009, China; zhangyw202110@163.com; 2 Beijing Key Laboratory of Millimeter Wave and Terahertz Technology, Beijing Institute of Technology, Beijing 100081, China; 3 Shanxi Key Laboratory of Advanced Semiconductor Optoelectronic Devices and Integrated Systems, Jincheng Research Institute of Opto-Mechatronics Industry, Jincheng 048000, China; yangleidtdx@163.com (L.Y.); lili@jcgjd.org.cn (L.L.)

In the original publication [1], the following funding information, “the Doctoral Start-up Funding of Shanxi Datong University (2023-B-64) and the Horizontal Cooperation Project of Shanxi Datong University (202512054)”, was not included. Correspondingly, the “Conflicts of Interest” section has been updated as follows: 

**Conflicts of Interest:** The authors declare that this study received funding from the Horizontal Cooperation Project of Shanxi Datong University (202512054). The funder was not involved in the study design, collection, analysis, interpretation of data, the writing of this article or the decision to submit it for publication.

The authors state that the scientific conclusions are unaffected. This correction was approved by the Academic Editor. The original publication has also been updated.

## References

[1] Zhang Y., Dong L., Hu W., Yang L., Li L. (2026). Research on the Composition and Manufacturing Technology of the Single-Eared Octagonal Gold Cup Unearthed from the Turki Mountain Tomb in Inner Mongolia. Materials.

